# New Year's Day 1915

**Published:** 1915-01-02

**Authors:** 


### The Hospital

The Workers' Newspaper of Administrative Medicine and Institutional Life, Administration, National Insurance and Health.

No. 1489, Vol. LVII. SATURDAY, JANUARY 2, 1915. PRICE ONE PENNY.

### NEW YEAR'S DAY 1915.

New Year's Day 1915 should be a day of remembrance for every member of the British race, whether residing near the battlefield, in the homeland, or in that portion of the world within the British Empire where his daily work has to be done. No members of the British race have ever before seen a New Year which has been so immediately preceded by months of cruel war, entailing destruction, wanton ill-treatment and outrage, lifelong injury and permanent ruin to so many human beings, the majority of them innocent victims to the lust and greed of their leaders and masters, or their enemies, or even of their own officers and fellow-countrymen. All war is cruel. The war of 1914 has been waged with barbarism and brutality absolutely unrestrained by any regard to Christian teaching. Indeed one great nation has claimed as a right that in the promotion of their ends atrocity, cruelty, murder, rapine, wanton and continuous destruction of property, art treasures, and innocent lives is a justifiable and sound policy for them as men of culture to practise. Since, however, a policy of cruelty and brutality, just because it deserves these names, is one which invariably recoils at last upon the heads of those who use it, we may be sure that without ourselves catching the infection, or raising clamour, mercy, and truth will be justified in the long run.

Our hospitals have contained much evidence of the barbarous use of forbidden missiles by the same contestants, for the Germans seem to glory in heaping up horrors, spreading suffering, and perpetuating cruelty on all defenceless people whenever they may penetrate into a neighbouring country to their own. There is not a right-feeling man or woman of any nation, apart from the Prussian nation, who will not, we believe, feel that the safety of the world demands that the nations who have practised the abominations to which we have just called attention must be overpowered, stricken down, and reduced to a condition where the combined peoples of Europe will make it impossible for them ever again to renew their criminal policy so long as the world endures. The German nation has shown scant respect for the Red Cross, and small regard for the wounded or the sick, or for those who treat and tend them. This the war has demonstrated to repletion, and yet the hospital and its attendants from the battlefield through all the stages to recovery or dissolution have pursued their Christ-like work with splendid courage and glorious success. So all hospital workers in every part of the British Empire, and in every portion of the Christian world, may welcome the New Year and offer their praises and thanksgivings to the great Giver of all good for the mercies which have come to the sufferers from the war through the hospitals everywhere. There has been a great awakening of charity, energy, and patriotism, and though these things should need no crisis like the present to arouse them, yet now that the crisis is here we may thankfully note their presence.

As a nation and Empire too the subjects of our King-Emperor, representing manifold nations and religions, who have spontaneously united against the cruel orgy of lustful greed and selfishness exhibited by the German War Lords and their rulers, may rejoice and thank God for the privilege of taking part in saving the world from the hideous degeneration and continuous misery which Germany plotted to impose on mankind. The year 1915 will mark a date in the present war from which it will be possible to forecast with certainty that the attempt to brutalise and destroy all that is best in human nature and the teachings of Christ will end not only in failure but in a revolution for good which must make the world a happier and better place for every innocent man and woman, to whatever nation he or she may belong, and wherever they may dwell. We offer to our readers the hope that they are able to spend New Year's Day 1915 in heartfelt thanksgiving, with an effort to do some real kindness to some one individual or living thing before the day is out. In giving expression to this hope we may remind our readers that it does not fall under the foolish old category of one good resolution or one good deed, since the voluntary hospital system itself teaches the ideal of personal service day in and day out, which is hourly affirmation of the above habit of mind.

